# Animal welfare: part of the solution, not part of the problem in the move toward achieving sustainable development in animal agriculture

**DOI:** 10.1093/af/vfaf009

**Published:** 2025-04-22

**Authors:** Linda J Keeling

**Affiliations:** Department of Applied Animal Science and Welfare, Swedish University of Agricultural Sciences, Uppsala, Sweden

**Keywords:** welfare, indicators, sustainable development, goals, synergies, future research

**Implications**

- How farm animals live their lives affects our lives. Animal welfare and sustainable development are inextricably linked.
- The lack of a systematic approach to assess the relationship between animal welfare and the Sustainable Development Goals has led to a focus on conflicts rather than synergies.
- A toolbox of animal welfare indicators exists, is continually expanding, and can be used in combination with other (e.g. environmental and sustainability) indicators.
- Scientists have an important role in identifying fruitful areas of research and ensuring valid and reliable monitoring of progress.

## A Brief Introduction to Animal Welfare Science—What’s the Problem?

Ethical concerns about the welfare of animals and the ambition to understand how animals experience their situation are key drivers in animal welfare research. It might have been sufficient to focus on individual animals and their welfare, perhaps even desirable when the scientific study of animal welfare first started, but this is no longer the case. Farm animals need to be taken seriously as a stakeholder in the future of our planet. A first step is raising awareness among scientists working with global challenges, that animal welfare is part of the solution and not a part of the problem.

Experimental studies have been crucial to our understanding of animal welfare and how to monitor it in commercial practice. Welfare assessment can now be part of a management tool for farmers, quality assurance schemes for supermarkets, and product labeling for consumers, as there are a growing number of valid, reliable, and feasible indicators of welfare on which to base decisions and monitor progress. However, this focus on animal products, and on satisfying customers’ concerns about the animal, has led to animal welfare science not connecting to other areas of concern, particularly those related to the environmental and societal pillars of sustainability. Failure to fully integrate the science of animal welfare into the sustainability framework may be a consequence of these animal welfare concerns only having been discussed in recent decades. Progress within the farm animal welfare field has also been slow as farmers and other actors along the supply chain criticize the field for adding costs to production. However, societal concerns necessitate that animal welfare science be integrated into the bigger picture of sustainable development. Buller et al. (2018) suggest five steps that need to happen if animal welfare is to become a significant and recognized component of the global drive toward a more sustainable agricultural development, while still retaining and building upon its robust and science base. They summarized them as integration (of farm animal welfare as a component of sustainability), articulation (by science and policy of the emergent concerns), representation (within the relevant international government structure), legitimation (by using robust and comparable standards) and innovation (in response to new scientific developments and policy challenges; Buller et al., 2018).

## The Bigger Picture

When referring to the bigger picture in the context of farm animal welfare, one usually thinks of the One Health and the related One Welfare concepts (Pinillos et al., 2016; OHHLEP, 2022). Nevertheless, there are concerns about our interactions with animals that, while perhaps not lost, are not yet fully explored within these concepts. The United Nations (UN) 2030 Agenda for Sustainability is a plan of action for people, planet, and prosperity, with 17 goals and 169 targets (United Nations, 2015).

The Sustainable Development Goals (SDGs) cover the three dimensions of sustainability (economic, social, and environmental) as well as additional institutional dimensions related to governance. They potentially cover all aspects of our interactions with animals, yet the SDGs are anthropocentric and animal welfare is not mentioned. The UN has realized this omission and in 2022 the UN Environmental Assembly adopted a resolution on the animal welfare–environment–sustainable development nexus (UNEP, 2022). Yet progress is painfully slow both for Agenda 2030 and for the inclusion of animal welfare. A serious problem, clearly presented in a recent high-profile commentary, is that actions toward SDGs are siloed and strategies unaligned (Nerini et al., 2024). While acknowledging the difficulties of extending the SDGs beyond 2030, these authors push “to shift the focus from negotiating over problems to delivering solutions, and to introducing strong enforcement mechanisms” (Nerini et al., 2024, p. 556). This statement applies equally well to animal welfare, where the focus also tends to be on problems, for example, conflicts with emissions and food security, as well as varying enforcement of legislation. It is as though animal welfare is regarded as a “luxury” on top of the “real” problems that we have with the sustainability of animal production today, rather than realizing that the welfare of an animal is an important component of the sustainability of that individual. When viewed in this way, improving animal welfare becomes a necessary and integral part of any solution.

## Animal Welfare Science—Part of the Solution

**Figure 1.**
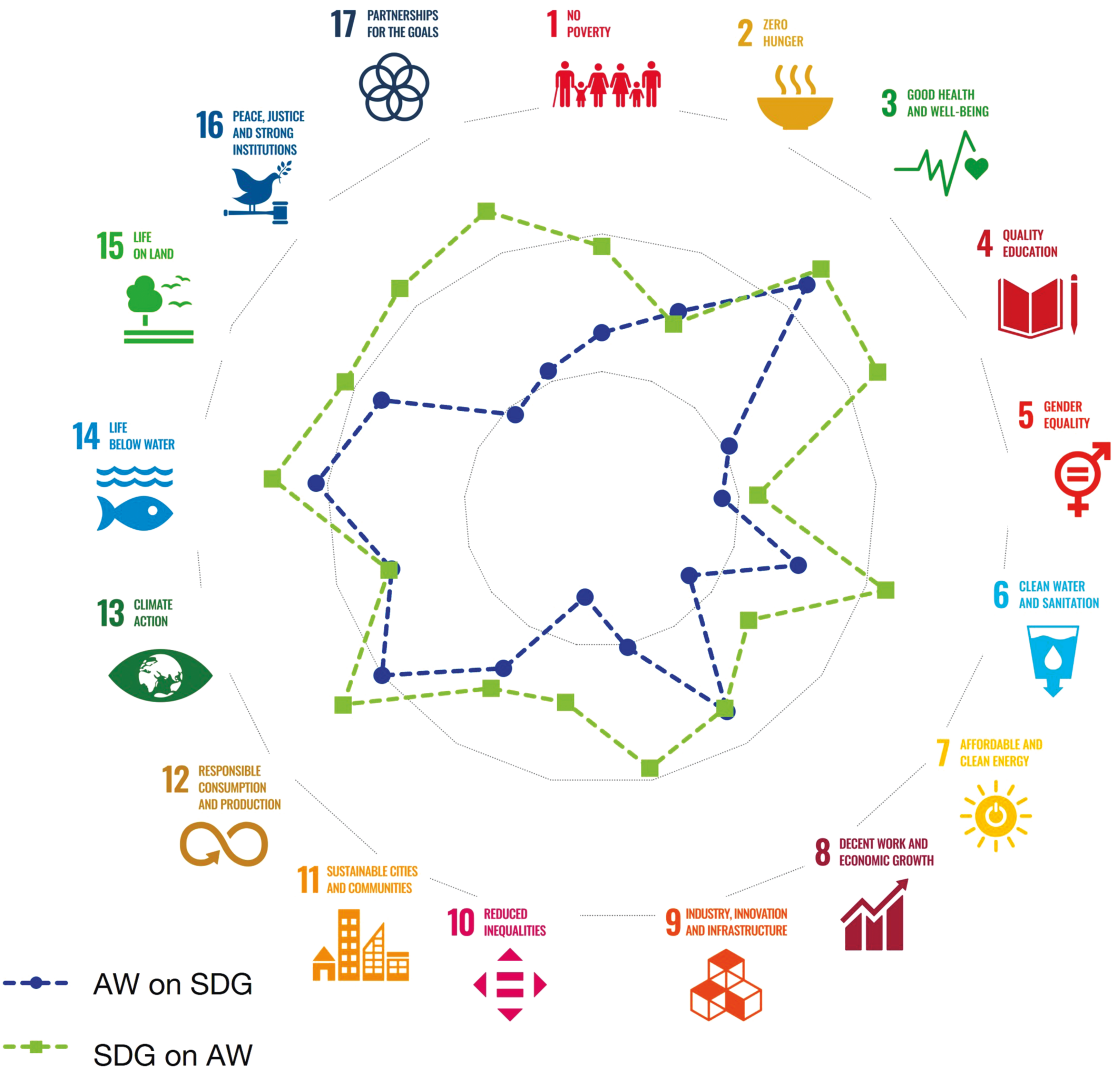
Radar plot of the mean rating scores for the impact of improving animal welfare (AW) on each Sustainable Development Goal (SDG) and the impact of achieving the SDG on AW. Although the original scale was −3 to +3, repeated studies have indicated that on average participants rate the impact as positive. The scale in this figure, therefore, is zero in the center of the circle and concentric circles representing scores of 1, 2, and 3, where a score of 3 is the outermost circle. Although the trend is for the impact of achieving the SDG on AW to be stronger (squares and the dashed line is mainly outermost. Green in online version) compared to the line representing the impact of improving AW on achieving the SDG (circles and dashed line mainly innermost. Blue in online version) there are SDGs where the lines overlap, implying that the impact is mutually beneficial. Figure modified from Keeling et al. (2022), which is an open-access article, distributed under the terms of the Creative Commons Attribution License. I acknowledge my coauthors and the journal Frontiers in Animal Science.

This final section is a brief presentation of a systematic approach we have used to explore the links between animal welfare and sustainable development, together with some potential next steps. Our methodology is summarized in Keeling et al. (2022) and involves asking participants to rate the links between the goal of improving animal welfare and each of the SDGs, in each direction, on a scale ranging between −3 (the two goals are incompatible and can not be achieved at the same time) to +3 (the two goals are fully linked and achieving one leads to the achievement of the other). Noticeable in our studies is that the average score for each animal welfare–SDG link is positive, meaning that participants see synergies rather than conflicts (Figure 1).

While at first surprising, there is recent evidence that this optimism may be part of a more general effect. A study to investigate the interlinkages between SDGs using the Global SDG database, which contains data from the 231 indicators within the UN monitoring framework, also found mostly synergies (positive correlations) rather than trade-offs (negative correlations) between indicators (Cling and Delecourt, 2022). Even in areas where we know, there are conflicts (pig production systems with low land use tend to have low greenhouse gas emissions, but high antimicrobial use and poor welfare, and vice versa) research findings suggest that “trade-offs […] are not inevitable,” that is, they may be avoidable (Bartlett et al., 2024, p. 1). A possible reason for the earlier negative attitude towards the inclusion of animal welfare in sustainable development (animal welfare as part of the problem rather than as part of the solution in animal agriculture) may, therefore, have been the lack of a systematic and holistic approach.

If animal welfare is to be a part of the solution, then scientists need to guide research and activities toward identifying the way forward. One approach to identify these may be to build upon our earlier methodology investigating the links between animal welfare and sustainable development (Keeling et al., 2022) using the following steps and modifications: (1) Repeat the original (−3 to +3) scoring exercise to rate the strengths of the links between animal welfare and each of the SDG, but now in specific farm animal contexts and using participants with relevant competences to reach a consensus score. (2) Visualize the results to identify areas of synergies and conflicts as a basis for decisions on where more research is needed and what policy decisions might be beneficial. (3) Prioritize working on synergies as a way to make progress toward solutions quickly, while giving researchers and innovators more time to work on mitigation strategies where there are conflicts. (4) Identify the appropriate animal-based welfare indicators that can be used in combination with existing relevant indicators (those already in the SDG framework) and potentially new indicators (e.g. from the three pillars of sustainability) for the selected farm animal context, as a holistic way to monitor overall progress toward goals.

In summary, animal welfare scientists have an important role to facilitate understanding that animal welfare and sustainability are linked, that animal welfare should be integrated into SDGs, and in nudging others to work on science-based synergies to accelerate progress.

## About the Author

**Linda Keeling** is Professor of Animal Welfare at the Swedish University of Agricultural Sciences, where she manages an active research group in the area of animal behavior and welfare. She received her PhD in Zoology from the University of Edinburgh, UK, and has worked previously for the Scottish Agricultural Colleges and at the University of Guelph in Canada. Her research involves investigating emotional states in animals (especially positive emotions), the effects of early experience on later behaviour, and the role of animal welfare in sustainable development. Among other activities, she is an Independent Expert member of the EU Platform on Animal Welfare and Editor for the Section of Animal Welfare and Policy in the journal Frontiers in Animal Science. She is an Honorary Doctor at the University of Linköping in Sweden and a Fellow of the Royal Swedish Academy of Forestry and Agriculture. **Corresponding author:**linda.keeling@slu.se
